# Combined Pre-Distortion and Censoring for Bandwidth-Efficient and Energy-Efficient Fusion of Spectrum Sensing Information

**DOI:** 10.3390/s17030654

**Published:** 2017-03-22

**Authors:** Guilherme Pedro Aquino, Dayan Adionel Guimarães, Luciano Leonel Mendes, Tales Cleber Pimenta

**Affiliations:** 1 National Institute of Telecommunications–Inatel, Av. João de Camargo, 510, 37540-000 Santa Rita do Sapucaí, Brazil; dayan@inatel.br (D.A.G.); lucianol@inatel.br (L.L.M.); 2 Federal University of Itajubá–Unifei, Av. BPS, 1303, 37500-903 Itajubá, Brazil; tales@unifei.edu.br

**Keywords:** bandwidth efficiency, censoring, cognitive radio, cooperative spectrum sensing, energy efficiency, fusion of sensor information, pre-distortion

## Abstract

This paper describes a novel scheme for the fusion of spectrum sensing information in cooperative spectrum sensing for cognitive radio applications. The scheme combines a spectrum-efficient, pre-distortion-based fusion strategy with an energy-efficient censoring-based fusion strategy to achieve the combined effect of reduction in bandwidth and power consumption during the transmissions of the local decisions to the fusion center. Expressions for computing the key performance metrics of the spectrum sensing of the proposed scheme are derived and validated by means of computer simulations. An extensive analysis of the overall energy efficiency is made, along with comparisons with reference strategies proposed in the literature. It is demonstrated that the proposed fusion scheme can outperform the energy efficiency attained by these reference strategies. Moreover, it attains approximately the same global decision performance of the best among these strategies.

## 1. Introduction

The cognitive radio (CR) concept [1] is considered one of the key enabling technologies of wireless communication systems, aiming at alleviating the problems of frequency spectrum scarcity and underutilization inherited by the current exponentially-growing service demand and the fixed allocation policy adopted around the world. The CR concept has been also listed as one of the key enabling technologies of the fifth-generation (5G) of mobile telecommunications networks [2]. One of the CR’s capabilities is to use parts of the frequency spectrum that are unoccupied by the primary network (the network to which is granted the prioritized use of that spectrum) in a given time and at a given location. Spectrum sensing [3] is the technique responsible for conferring this ability to the CRs. By applying this technique, a CR-equipped secondary user (SU) can detect the vacant bands (the so-called spectral holes or whitespaces) and use them opportunistically without causing any harmful interference to the primary user (PU) radios.

The performance of a spectrum sensing technique is often assessed by means of receiver operating characteristic (ROC) curves, which trade the probability of detection and the probability of false alarm, which are considered the key performance metrics. The former is the probability of declaring the presence of the PU signal under the hypothesis that it is really present; the latter is the probability of declaring the presence of the PU signal under the hypothesis that it is in fact absent. Higher probabilities of detection are translated into low probabilities of causing interference to the primary network; low probabilities of false alarm translate into higher chances of opportunistic transmissions in the secondary networks, thus increasing the data throughput.

The performance of spectrum sensing may be seriously degraded due to channel and receiver noise, multi-path fading, shadowing and hidden terminal problem [3], inducing CRs to make inaccurate decisions about the occupancy state of the sensed band. Cooperative spectrum sensing (CSS) is capable of alleviating the effect of these impairments by exploring spatial diversity [4], thus increasing the reliability of the decision on the occupation of the sensed channel. In CSS, a number of secondary users jointly perform spectrum sensing and send the associated measurements or local decisions to a fusion center (FC), where a global decision on the channel occupancy is performed by combining the information provided by the SUs. In traditional CSS, there is a dedicated report channel for each SU to transmit its information to the FC, normally using some form of orthogonal multiple access, as for instance traditional time division- or frequency division-based ones.

Although increasing the number of SUs contributes to an improvement in the global decision accuracy, more resources, such as bandwidth and energy, are needed. Thus, orthogonal transmissions of sensing results to the FC combined with an increased number of cooperating CRs are two factors that increase the resource expenditure in CSS schemes.

### 1.1. Related Works

Several techniques have been proposed to reduce the resource utilization in CSS schemes, as described in the sequel.

In [5], the energy efficiency of a soft-decision fusion strategy is improved by mapping the quantization levels of the sensing information into the position of a single bit in different time slots of the frame structure, instead of mapping these levels into different binary words. In [6], the global decision at the FC can be anticipated, preventing some SUs from sending their sensing information, thus reducing the average number of bits transmitted through the report channel and increasing the energy efficiency. The authors of [7,8] investigate the effect of the sensing time, the number of cognitive radios, the detection threshold and the primary user spectrum occupancy rate in order to find the optimum sensing parameters that maximize the energy efficiency of the CSS network.

Also aiming at increasing energy efficiency, a number of strategies adopt the concept of letting a subset of the SUs active to sense and (or) report their decisions to the FC, while the other SUs remain in sleep mode. This concept falls into the broad category of node selection [9], in which the following contributions are worth highlighting: In [10,11], only the users with high reliability are allowed to report their local binary decisions to the FC. A cluster-based CSS approach is explored in [12,13,14,15,16], where the SUs are grouped into different clusters, and only the SU with the best report channel signal-to-noise ratio (SNR) is responsible for transmitting the cluster decision to the FC. A censored, truncated method with sequential spectrum sensing is proposed in [17], where an SU is forced to stop sensing when the accumulated energy of the collected samples falls into a certain region; then, its local decision is transmitted to the FC. Otherwise, if this SU does not detect a level of energy in the specified region, no bits are sent to the FC. In [18] is proposed a scheme in which a single SU broadcasts its local decision. The remaining SUs object or agree with the first SU. The SUs that have a decision different from the announced one send the objection information to the FC, whereas the agreeing SUs remain silent during the report time. In [19] is proposed a channel quality-based activation rule that chooses some SUs to be active during the sensing and report phases. In [20,21], a sequential cooperative spectrum sensing (SCSS) scheme is explored. In SCSS, the FC coordinates the report of local decisions in the secondary network, choosing the SUs that report their local sensing information. These SUs then randomly send their decisions until the condition required to make a global decision is satisfied at the FC.

In spite of promoting increased energy efficiencies, all of the above contributions do not care about improving the bandwidth efficiency, since they assume the traditional CSS fusion scheme in which orthogonal report channels are used.

Aiming at saving the report channel bandwidth, in [22,23], a cooperative spectrum sensing without a dedicated report channel is proposed. In this proposal, the SUs send their local decisions to the FC using licensed PU channels. The authors shows how to mitigate the interference caused by the SUs in the PU devices. Results show that it is possible to save the dedicated control channel resources without sacrificing the sensing performance and PU operation. In [24], a bandwidth-efficient method is proposed for reducing the amount of report data in a distributed CSS. In this scheme, each SU shares the local decision with its neighbor, sequentially and in a ring basis, and the last SU makes the global decision about the PU signal presence. In [25], a sequential test is introduced at each SU for the local decision report. Each SU reports its decision only after having enough confidence. Therefore, each PU reports its decision at a different time, decreasing the necessary bandwidth of the control channel. Despite promoting bandwidth savings, the approaches described in this paragraph do not provide any means for reducing and analyzing the energy consumption.

In [26], the author introduces a new CSS scheme in which the cooperating CRs report their local hard decisions on the same carrier frequency, at the same time. Therefore, during the report phase, the received signal at the FC will be equal to the non-coherent sum of the signals carrying all local decisions. In other words, due to the simultaneous transmissions of the local decisions, a self-interference among the received signals at the FC intentionally arises, but bandwidth resources are saved. A global decision rule based on the maximum likelihood (ML) criterion is also provided in [26], along with equations for computing the global probabilities of detection and false alarm over the additive white Gaussian noise (AWGN) channel. This decision rule works under the assumption that the channel gains between the SUs and the FC are known to the FC. Subsequently, in [27], the authors extend the work of [26] and give expressions for computing the global probabilities of detection and false alarm over real-valued Rayleigh report channels. Comments are given in [27] regarding the complexity of finding similar equations for the scenario of complex-valued channel gains.

A resource-efficient fusion with pre-compensated transmissions was proposed in [28], targeting a two-fold goal: (i) transform the intricate problem of global decision over a complex-valued channel into a simple one-dimensional threshold-based decision problem; (ii) allow for the derivation of expressions for computing the approximate values of the global probabilities of false alarm and detection. The term pre-compensated transmissions stems from the fact that channel estimates based on sounding signals broadcast from the FC to the SUs are used at the SUs for pre-distorting the transmitted signals so as to compensate for the report channel phase rotation and to partially compensate for the channel gain.

Although the pre-compensation (or pre-distortion) strategy adopted in [28] promotes a significant reduction in the system implementation complexity when compared with the original one proposed in [26], saving spectrum resources likewise in [26], it still suffers from the high energy consumption problem of traditional CSS schemes during the report phase, a problem that also happens in the original approach of [26]. It is desirable to save the energy during this phase, for instance to use it in signal processing and data transmission tasks.

Recently, [29] proposed a censoring report rule to be applied to the scheme of [26], aiming at reducing the energy consumption of the SUs during the report of local decisions to the FC. In this rule, which falls into the node selection approach, only the SUs declaring the presence of the PU signal are allowed to transmit their local decisions to the FC. According to the results shown in [29], the censoring rule can promote a considerable increase in the energy efficiency of the fusion rule proposed in [26]. However, the censoring rule of [29] can be applied only when AWGN report channels are assumed.

### 1.2. Contributions and Structure of the Paper

In this paper is proposed the combination of a bandwidth-efficient, pre-distortion-based fusion scheme with an energy-efficient censoring-based fusion scheme to achieve the joint effect of reduction in bandwidth occupation and energy expenditure during the report phase of the cooperative spectrum sensing over complex fading report channels. Expressions for computing the key performance metrics of the spectrum sensing of the new fusion scheme are derived and validated by means of computer simulations. An extensive analysis of its overall energy efficiency is also made, as well as comparisons with the schemes suggested in [26,28]. Our scheme significantly reduces the global decision complexity when compared to [26] and achieves performances that closely approximate those reported in [28]. Moreover, substantially higher energy efficiencies are achieved by our scheme with respect to [26,28]. It is worthy highlighting that [26,28] were selected for comparisons because they represent the state-of-the-art-related approaches for simultaneously saving bandwidth and energy during the report phase in CSS schemes based on decision fusion.

The remainder of this paper is organized as follows. The two system models adopted as references for background and comparisons are discussed in Section 2. Section 3 is devoted to the proposed energy-efficient fusion scheme. The analysis of energy consumption is made in Section 4. Numerical results and discussions are provided in Section 5. Finally, Section 6 concludes the paper.

## 2. Reference System Models

The cooperative spectrum sensing consists of *M* secondary users that transmit their local decisions to the fusion center using a binary phase-shift keying (BPSK) modulation [30]. Those transmissions occur at the same time and on the same carrier frequency, thus leading to a more efficient use of the report channel bandwidth of the cognitive secondary network. The fusion scheme of [26] is considered the original reference for such fusion principle. The fusion scheme of [28] is the other reference that works under the same principle. These two schemes are addressed in this section to set the notation, the background and the corresponding system models.

### 2.1. Original Fusion Scheme

In [26], the *k*-th secondary user, k=1,⋯,M, performs local spectrum sensing that results in a binary decision $m_{k}=0$ representing the hypothesis $H_{0}$ (absence of the PU signal) or $m_{k}$ = 1 representing the hypothesis $H_{1}$ (presence of the PU signal) in the bandwidth of interest. The local decision is mapped into a BPSK symbol at each SU, yielding $s_{k}=\left(2m_{k}-1\right)\sqrt{\xi }$ in baseband representation [30], where *ξ* is the average energy per transmitted symbol. Assuming that the report channel between the *k*-th SU and the FC has a complex gain $h_{k}$ that is kept unchanged during the report phase, the received signal sample at the FC is given by the incoherent sum of all transmitted BPSK symbols plus noise, that is,

(1) $$r=\sum_{k=1}^{M}h_{k}s_{k}+n,$$

where *n* is the zero-mean AWGN sample with variance $\sigma^{2}$ and unilateral power spectral density $N_{0}=2\sigma^{2}$ watts/hertz.

It is assumed in [26] that the FC knows the complex report channel gain vector $h={\left[h_{1},h_{2},\cdots ,h_{M}\right]}^{T}$, where the superscript T denotes vector transposition, allowing the classification of the noiseless received samples into the sets $D_{0}$ and $D_{1}$, which are the sets associated with the local decision vector $s={\left[s_{1},s_{2},\cdots ,s_{M}\right]}^{T}$ that would lead to the choice of $H_{0}$ and $H_{1}$, respectively, under the *K*-out-of-*M* decision fusion rule. In mathematical terms,

(2) $$\begin{array}{c} D_{0}=\left\{s\;\;|\;\;\sum_{k=1}^{M}m_{k}<K\right\},\\ D_{1}=\left\{s\;\;|\;\;\sum_{k=1}^{M}m_{k}\geq K\right\}. \end{array}$$

The global decision rule proposed in [26] determines that FC will decide in favor of $H_{1}$ if:

(3) $$\sum_{s\in D_{1}}\exp\left\{-\frac{{\left(r-h^{T}s\right)}^{2}}{2\sigma^{2}}\right\}\geq \sum_{s\in D_{0}}\exp\left\{-\frac{{\left(r-h^{T}s\right)}^{2}}{2\sigma^{2}}\right\},$$

and otherwise will decide in favor of $H_{0}$.

It is important to bear in mind that the report channel gains are needed in the decision rule (3). According to [26], one could resort to sounding signals transmitted in the up-link direction (from the SUs to the FC) to estimate these gains. However, this would require a large amount of resources of the cognitive network, since those sounding signals would have to be sent over orthogonal channels corresponding to all links between the SUs and the FC.

Expressions for computing the probabilities of detection and false alarm are given in [26], assuming AWGN report channels. Simulation results are presented for AWGN and Rayleigh fading report channels. Complementary expressions are provided in [27] for computing the global probabilities of detection and false alarm, assuming report channels with real-valued time-varying gains.

### 2.2. Fusion with Pre-Distorted Transmissions

The intricate problem of the global decision over complex-valued report channels as suggested in [26] is transformed into a simple one-dimensional threshold-based decision problem in [28], resulting in considerable reduction of system complexity and, in some situations, better performances when compared to [26]. The main idea of [28] was to shift the channel estimation task from the FC to the SUs so that channel estimates computed by each SU are used to pre-distort the transmitted signals in order to compensate for the report channel phase rotations and to partially compensate for the magnitude gains. Another positive side-effect of this idea is the reduction in the amount of resources needed to send sounding signals for the purpose of channel estimation: a single broadcast channel could be used in the down-link (from the FC to the SUs), instead of the multiple orthogonal up-link channels needed in [26].

The *k*-th complex report channel gain is defined as $h_{k}=\alpha_{k}e^{j\theta_{k}}$, where $\alpha_{k}$ and $\theta_{k}$ are the magnitude gain and phase rotation, respectively. Before reporting its local decision, each SU could pre-compensate for both the channel phase rotation and the magnitude gain. The pre-compensation of the phase rotation brings no negative side-effect. However, when $\alpha_{k}$ is too small, pre-compensation of magnitude gain would result in prohibitively high transmission levels, thus significantly increasing the peak-to-average power ratio (PAPR) of the transmitted signal. High PAPR imposes strong restrictions to the design of the power amplifiers for the SUs and causes large peaks of energy consumption.

A simple way of reducing the PAPR is by means of signal clipping, which limits the maximum amplitude of the transmitted signal. Then, as a result of total phase rotation pre-compensation and partial (with clipping) gain magnitude pre-compensation, a baseband BPSK symbol transmitted by the *k*-th SU can be written as:

(4) $$s_{k}=\left(2m_{k}-1\right)\min\left(\frac{1}{\alpha_{k}},C\right)e^{-j\theta_{k}}\sqrt{\xi },$$

where *C* is the clipping threshold. This threshold defines the maximum transmitted signal amplitude for preventing prohibitively high instantaneous transmission powers that would result from the attempt to compensate for very low channel gains $\alpha_{k}$, k=1,⋯,M. In practice, the value of *C* is chosen according to a trade-off between the desired energy efficiency and the performance of the CSS scheme, as demonstrated later on in Section 5.

Since the *M* secondary users transmit their local decisions at the same time and in the same carrier frequency, a received signal sample at the FC is now given by a coherent sum, that is,

(5) $$r=\sum_{k=1}^{M}\left(2m_{k}-1\right)\alpha_{k}\min\left(\frac{1}{\alpha_{k}},C\right)\sqrt{\xi }+n.$$

Observe that the received signal samples are real-valued due to the total pre-compensation of the report channel phase rotations made at the SUs. When $C>1/\alpha_{k}$, the noiseless received samples ρ=r−n at the FC follow a binomial distribution with M+1 values. These values can be associated with the M+1 points of a one-dimensional constellation and can be classified into two different sets. The constellation points associated with $H_{1}$ are $\left\{\left(2K-M\right)\sqrt{\xi },\cdots ,M\sqrt{\xi }\right\}$, and the points associated with $H_{0}$ are $\left\{-M\sqrt{\xi },\cdots ,\left(2K-M-2\right)\sqrt{\xi }\right\}$. As the clipping threshold *C* is reduced in order to reduce the PAPR of the transmitted signal, the clipping effect starts to be noticed in the probability density function (PDF) of *ρ*, which now also exhibits a continuous part. According to [28], for some values of *C*, this continuous part corresponds approximately to a sum of weighted, shifted and scaled two-sided truncated Rayleigh PDF whose area is directly proportional to the probability of clipping.

The approach of pre-distortion simplifies the decision regions, allowing the FC to make the global decision based on a simple comparison of the received sample with a threshold given by [28]:

(6) $$\lambda =\left(2K-M-1\right)\sqrt{\xi }.$$

Accurate expressions for approximate computation of the global probabilities of detection and false alarm of the fusion with the pre-compensated transmissions scheme are presented in [28]. Moreover, it is shown in [28] that this fusion scheme can produce large performance improvements over the one proposed in [26], with reduced complexity due to a simpler one-dimensional threshold-based decision rule. In spite of these advantages, the fusion with pre-compensated transmissions scheme still preserves the negative characteristic of CSS systems regarding the energy consumption.

## 3. Proposed Fusion with Pre-Distorted Transmissions and Censoring

Aiming at promoting bandwidth efficiency, reduction of the energy consumption during the report phase and simple decision rule at the FC, here, the pre-distortion technique is combined with a censoring strategy and applied to the simultaneous transmission scheme proposed in [26].

### 3.1. System Model

The whole process of CSS can be divided into three phases: (i) local spectrum sensing; (ii) local decision transmission (report phase); and (iii) global decision at the FC. In the first phase, the activity of the PU is observed by each SU by means of any of the sensing technique described in the literature, for instance energy detection, matched filter detection, cyclostationary feature detection, or eigenvalue-based detection [3]. During the report phase, a number M≤M of SUs that decided on the presence of the PU signal transmit their local decision to the FC. The remaining M−M transmitters keep turned-off, thus saving energy. In the last phase, the FC makes the global decision upon spectrum use based on the local decisions received from the M SUs.

Assuming that $h_{k}=\alpha_{k}e^{-j\theta_{k}}$ is known by the *k*-th SU through a proper channel estimation process, each SU has its decision $m_{k}=0$ translated into a turned-off transmitter (as a result of censoring) and $m_{k}=1$ mapped into a complex symbol with energy *E*, pre-compensated for the channel phase rotation and partially pre-compensated (i.e., pre-compensated with clipping) for the channel magnitude gain. The baseband representation of the SU signal activity is:

(7) $$s_{k}=m_{k}\sqrt{E}e^{-j\theta_{k}}\min\left(\frac{1}{\alpha_{k}},C\right),$$

and the received sample at the FC is then:

(8) $$r=\sum_{k=1}^{M}m_{k}\sqrt{E}\alpha_{k}\min\left(\frac{1}{\alpha_{k}},C\right)+n,$$

where, likewise in (4), $1/\alpha_{k}$ represents the gain applied to the transmitted signal in order to compensate for the fading channel gain.

According to the proposed censoring rule, the transmission of a local decision on $H_{1}$ occurs with probability $p=\mathrm{Pr}[m_{k}=1]$, which is given by:

(9) $$p=P_{H_{0}}P_{\mathrm{FA},\mathrm{SU}}+P_{H_{1}}P_{D,\mathrm{SU}},$$

where $P_{H_{1}}$ and $P_{H_{0}}=1-P_{H_{1}}$ are the probabilities that the PU transmitter is on and off, respectively, and $P_{D,\mathrm{SU}}$ and $P_{\mathrm{FA},\mathrm{SU}}$ are the local probabilities of detection and false alarm at the SUs, respectively. Without loss of generality and for the sake of simplicity, it is assumed that each SU performs independent sensing using energy detection (ED) over AWGN channels between the PU transmitter and the SUs (recall that the proposed fusion strategy refers to the transmission of the SUs’ decisions to the FC, thus being applicable to any spectrum sensing technique and PU-SUs channel model). In this case, the local probabilities of detection and false alarm are approximately given by [31] (pp. 18–19):

(10) $$P_{D,\mathrm{SU}}=Q\left(\frac{\zeta -N(\sigma_{w}^{2}+\sigma_{x}^{2})}{\sqrt{2N{\left(\sigma_{w}^{2}+\sigma_{x}^{2}\right)}^{2}}}\right),$$

(11) $$P_{\mathrm{FA},\mathrm{SU}}=Q\left(\frac{\zeta -N\sigma_{w}^{2}}{\sqrt{2N\sigma_{w}^{4}}}\right),$$

where *ζ* is the ED’s decision threshold, *N* is the number of received signal samples collected by each SU, $\sigma_{x}^{2}$ is the power of the zero-mean PU signal, $\sigma_{w}^{2}$ is the noise power at the input of the SU’s receivers and Q(·) is the Gaussian tail probability function.

From (7), the event of signal clipping occurs if the hypothesis $H_{1}$ is declared by a given SU, i.e., $m_{k}=1$, and if $1/\alpha_{k}>C$ (or equivalently if $\alpha_{k}\leq 1/C$). By noticing that these two conditions are independent of each other, the probability of clipping can be written as:

(12) $$p_{\mathrm{clip}}=p\mathrm{Pr}\left[\alpha_{k}\leq 1/C\right],\;\forall k.$$

Hereafter, it is assumed that the report channels are flat and slow (constant during a report transmission) Rayleigh fading channels, i.e., $\alpha_{k}$ is Rayleigh-distributed and $\theta_{k}$ is uniformly-distributed in (0,2π], with independent realizations from one report round to the next. In this case, the probability of clipping can be expressed as:

(13) $$\begin{array}{cc} p_{\mathrm{clip}} & =p\int_{0}^{1/C}\frac{2z}{\Omega }\exp\left(-\frac{z^{2}}{\Omega }\right)dz\\ & =p\left[1-\exp\left(-\frac{1}{C^{2}\Omega }\right)\right], \end{array}$$

where Ω is the second moment of the Rayleigh fading magnitude [32].

### 3.2. PDF of the Noiseless Received Samples Due to a Single SU

The contribution of a single SU signal to the noiseless received signal sample at the FC is defined as:

(14) $$\rho_{1}=m_{k}\sqrt{E}\alpha_{k}\min\left(\frac{1}{\alpha_{k}},C\right),\;\forall k.$$

In the absence of clipping, $\rho_{1}$ follows a Bernoulli distribution for the values $\rho_{1}=\sqrt{E}$ and $\rho_{1}=0$ that occur with probabilities *p* and (1−p), respectively. When clipping occurs, it turns out that values of $\rho_{1}$, for $0<\rho_{1}<\sqrt{E}$, also appear with probability $p_{\mathrm{clip}}$. From (14), if clipping occurs, then $\min\left(\frac{1}{\alpha_{k}},C\right)=C$, and the distribution of $\rho_{1}$ is the distribution of $\sqrt{E}\alpha_{k}C$ over the open support $\left(0,\sqrt{E}\right)$, which corresponds to a scaled and truncated Rayleigh PDF. Therefore, the overall PDF of $\rho_{1}$ can be expressed in terms of the combination of a discrete and a continuous part, yielding:

(15) $$f\left(\rho_{1}\right)=\left(1-p\right)\delta \left(\rho_{1}\right)+\left(p-p_{\mathrm{clip}}\right)\delta \left(\rho_{1}-\sqrt{E}\right)+\frac{2\rho_{1}p\left[1-u(\rho_{1}-\sqrt{E})\right]}{\Omega C^{2}E}\exp\left(-\frac{{\rho_{1}}^{2}}{\Omega C^{2}E}\right),$$

where δ(·) is the Dirac-delta function and u(·) is the unit-step function. Notice in (15) that the probability of the value $\rho_{1}=\sqrt{E}$ is decreased from *p* to $\left(p-p_{\mathrm{clip}}\right)$ by the action of clipping. The difference, $p_{\mathrm{clip}}$, is transferred to the continuous part whose area is obviously $p_{\mathrm{clip}}$.

**Figure 1:**
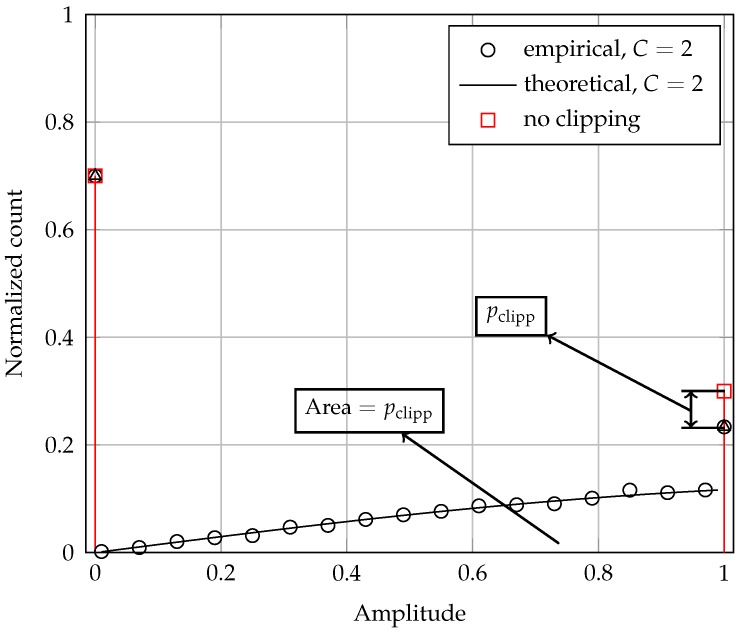
Probability density function of the noiseless received signal samples due to a single SU for p=0.3, C=2 and E=1. The situation of no clipping is also shown for reference.

A sketch of the theoretical PDF (15) can be seen in Figure 1, along with the corresponding empirical PDF obtained from a computer generation of 700,000 values of $\rho_{1}$. The close agreement between these two PDFs is readily observed. The PDF of $\rho_{1}$ in the absence of clipping is also shown for reference, especially to illustrate the reduction of $p_{\mathrm{clip}}$ on $f(\sqrt{E})$ due to clipping and the transference of this reduction to the area of the continuous part that appears when clipping occurs. To plot this figure, the system parameters were arbitrarily chosen as p=0.3, C=2 and E=1.

### 3.3. PDF of the Noiseless Received Samples Due to All SUs

The analysis is now moved to the situation in which the received signal at the FC results from the coherent sum of the M≤M signals transmitted by the SUs that decided in favor of the presence of a PU signal, according to the censored report rule. Therefore, the noiseless real-valued received sample at the FC becomes:

(16) $$\rho =\sum_{k=1}^{M}m_{k}\sqrt{E}\alpha_{k}\min\left(\frac{1}{\alpha_{k}},C\right).$$

Assuming independence among the signals received from the SUs, the PDF f(ρ) could be derived as the convolution of *M* PDFs described by (15) or by applying the inverse Fourier transform to the product of the *M* characteristic functions of $f(\rho_{1})$. However, such derivation seems to be mathematically intractable. Nonetheless, f(ρ) can be approximated, as described in the sequel.

Let the PDF (15) be decomposed into its discrete part $f^{d}\left(\rho_{1}\right)$ and its continuous part $f^{c}\left(\rho_{1}\right)$, such that $f\left(\rho_{1}\right)=f^{d}\left(\rho_{1}\right)+f^{c}\left(\rho_{1}\right)$. For the sake of simplicity, assume for a while that M=2, a case in which the PDF of *ρ* becomes:

(17) $$\begin{array}{cc} f(\rho ) & =f\left(\rho_{1}\right)*f\left(\rho_{1}\right)\\ & =\left[f^{d}\left(\rho_{1}\right)+f^{c}\left(\rho_{1}\right)\right]*\left[f^{d}\left(\rho_{1}\right)+f^{c}\left(\rho_{1}\right)\right]\\ & =f^{d}\left(\rho_{1}\right)*f^{d}\left(\rho_{1}\right)+2f^{d}\left(\rho_{1}\right)*f^{c}\left(\rho_{1}\right)+f^{c}\left(\rho_{1}\right)*f^{c}\left(\rho_{1}\right), \end{array}$$

where the properties of commutativity and distributivity of the convolution operation represented by ∗ were applied. If clipping is sufficiently small, the terms $f^{d}\left(\rho_{1}\right)*f^{d}\left(\rho_{1}\right)+2f^{d}\left(\rho_{1}\right)*f^{c}\left(\rho_{1}\right)$ will be considerably larger than $f^{c}\left(\rho_{1}\right)*f^{c}\left(\rho_{1}\right)$, since the probabilities associated with the discrete part of $f(\rho_{1})$ will be considerably larger than the area $p_{\mathrm{clip}}$ of its continuous part. The same reasoning extends to M>2.

The approximation adopted here is that the discrete part $f^{d}\left(\rho \right)$ of f(ρ) is given by the multiple self-convolution (*M* times) of $f(\rho_{1})$ and that the continuous part $f^{c}\left(\rho \right)$ of f(ρ) is approximately given by properly weighted replications of shifted versions of $f^{c}\left(\rho_{1}\right)$. These replications are due to the convolution between discrete and continuous parts, as exemplified in (17) by the term $2f^{d}\left(\rho_{1}\right)*f^{c}\left(\rho_{1}\right)$. The smaller the number *M* of SUs and the larger the clipping threshold *C*, the better the approximation will be.

By direct implication of the relation between the convolution operation and the binomial theorem, the *i*-th element of the multiple self-convolution of the sequence $a=[a_{0},a_{1}]$ with real or complex elements $a_{0}$ and $a_{1}$ can be computed as:

(18) ci(M)=Mia1ia0M−i,i=0…M.

Then, the probabilities in $f^{d}\left(\rho \right)$ can be determined from the *M* self-convolutions of the probability values associated with $f^{d}\left(\rho_{1}\right)$, which are (1−p) and $\left(p-p_{\mathrm{clip}}\right)$, yielding:

(19) fd(ρ)=∑i=0MMi(p−pclip)i(1−p)M−iδ(ρ−iE).

The continuous part of f(ρ) approximates replications of the continuous part in (15), under different shifts and weights, yielding:

(20) $$f^{c}\left(\rho \right)=\sum_{i=1}^{M}\left[1-u(\rho -i\sqrt{E})\right]\left[\frac{2(\rho -i+1)}{\Omega C^{2}E}\exp\left(-\frac{{\left(\rho -i+1\right)}^{2}}{\Omega C^{2}E}\right)\right]\left[\frac{pA_{i}\left(p\right)}{p_{\mathrm{clip}}}\right],$$

where $A_{i}\left(p\right)$ is the area of the *i*-th replication of the truncated Rayleigh distribution, which is given by:

(21) Ai(p)=Mi(1−p)M−ipi−(p−pclip)i.

Therefore, the approximated PDF for the noiseless samples at the FC can be written as:

(22) $$f\left(\rho \right)=f^{d}\left(\rho \right)+f^{c}\left(\rho \right).$$

**Figure 2:**
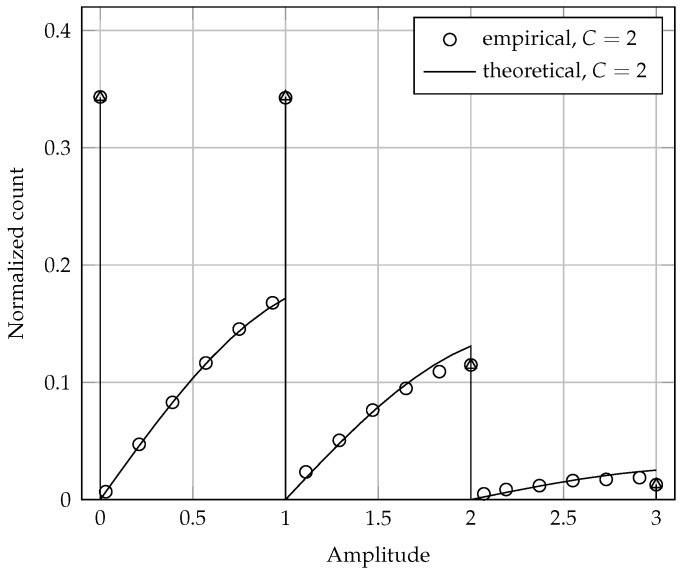
Probability density function of the noiseless received signal samples at the FC, for M=3, p=0.3, C=2 and E=1.

This PDF is illustrated in Figure 2 for M=3, p=0.3, C=2 and E=1. It can be seen that the approximated (theoretical) PDF is in close agreement with the actual (empirical) PDF of the noiseless received signal samples. An analysis about the adherence between the theoretical and empirical results is made in Section 5.3.

The influence of the clipping threshold *C* on the shapes of Figure 1 and Figure 2 occurs as follows: as *C* increases, the effect of clipping becomes less severe, reducing the areas of the continuous parts in these PDFs. In the limit C→∞, these continuous parts vanish, only the discrete parts remaining that represent the PDFs of the corresponding noiseless and distortionless symbols received at the FC.

### 3.4. Global Probabilities of Detection and False Alarm

From (19) or from Figure 2, one can notice the prevalence of the noiseless received signal samples at $\rho =i\sqrt{E}$, i=0,⋯,M. Under the *K*-out-of-*M* rule, the indexes i<K correspond to the global choice in favor of $H_{0}$, whilst the indexes i≥K correspond to the global choice for $H_{1}$. This suggests a decision threshold *λ* somewhere in-between $\left(K-1\right)\sqrt{E}$ and $K\sqrt{E}$, meaning that the FC will choose $H_{1}$ if ρ≥λ and will choose $H_{0}$ otherwise.

Finally, taking into account the addition of the Gaussian noise that is present at the FC receiver input, the average global probability of detection, $P_{D,\mathrm{FC}}$, and false alarm, $P_{\mathrm{FA},\mathrm{FC}}$, can be computed as the corresponding probabilities of detection and false alarm conditioned on a given value of *ρ*, averaged over all possible values of *ρ*, yielding:

(23) $$P_{D,\mathrm{FC}}=\sum_{i=0}^{M}Q\left(\frac{\lambda -i\sqrt{E}}{\sqrt{2\gamma_{\mathrm{FC}}}}\right)f^{d}\left(\rho |p=P_{D,\mathrm{SU}}\right)+\int_{0}^{M}Q\left(\frac{\lambda -\rho }{\sqrt{2\gamma_{\mathrm{FC}}}}\right)f^{c}\left(\rho |p=P_{D,\mathrm{SU}}\right)d\rho ,$$

(24) $$P_{\mathrm{FA},\mathrm{FC}}=\sum_{i=0}^{M}Q\left(\frac{\lambda -i\sqrt{E}}{\sqrt{2\gamma_{\mathrm{FC}}}}\right)f^{d}\left(\rho |p=P_{\mathrm{FA},\mathrm{SU}}\right)+\int_{0}^{M}Q\left(\frac{\lambda -\rho }{\sqrt{2\gamma_{\mathrm{FC}}}}\right)f^{c}\left(\rho |p=P_{\mathrm{FA},\mathrm{SU}}\right)d\rho ,$$

where $\gamma_{\mathrm{FC}}$ is the average SNR (signal-to-noise ratio) at the FC receiver input.

The dependence of the Equations (19) and (20) on $p=P_{D,\mathrm{SU}}$ or $p=P_{\mathrm{FA},\mathrm{SU}}$ can be noticed, meaning that the global optimum decision threshold depends on the knowledge of the instantaneous performance of the spectrum sensing at the SUs, information that normally is not available in practice. Here, simplified sub-optimal thresholds are proposed for the most widely-used *K*-out-of-*M* rules, which are the OR (K=1), the AND (K=M) and the majority-voting (K=⌈M/2⌉), as follows:

(25) $$\lambda =\left\{\begin{array}{c} \sqrt{E}\;,\;\mathrm{for}\;\mathrm{the}\;\mathrm{OR}\;\mathrm{rule},\\ \left(M-1\right)\sqrt{E}\;,\;\mathrm{for}\;\mathrm{the}\;\mathrm{AND}\;\mathrm{rule},\;\mathrm{and}\\ \left(\left\lceil M/2\right\rceil -1/2\right)\sqrt{E}\;,\;\mathrm{for}\;\mathrm{the}\;\mathrm{majority}-\mathrm{voting}\;\mathrm{rule}. \end{array}\right.$$

It was found empirically that these thresholds do not produce significant performance degradation of the spectrum sensing when compared with the scheme of [28], which is superior to the original one proposed in [26], yet avoiding the need for knowing $P_{D,\mathrm{SU}}$ and $P_{\mathrm{FA},\mathrm{SU}}$. We postpone the verification of these statements to Section 5, where numerical results are presented and discussed.

## 4. Energy Consumption Analysis

For the purpose of energy consumption analysis, the SU operation time-frame can be divided into three parts: (i) local sensing; (ii) report; and (iii) decision and opportunistic data transmission. In the first part, all SUs perform spectrum sensing and decide on the presence or absence of the PU signal. In the second part, the SUs transmit their decisions to the FC. In the third part, the FC makes the global decision about the state of the PU transmitter, and if it is declared off, the SUs are allowed to start their opportunistic communication. The SUs remain silent if the PU transmitter is declared on.

Following [29], the average energy consumption per frame in a CSS scheme is given by:

(26) $$E_{\mathrm{css}}=ME_{\mathrm{LS}}+ME_{R}+P_{\mathrm{free}}E_{T},$$

where $E_{\mathrm{LS}}$, $E_{R}$ and $E_{T}$ are the energy consumed by a single SU during local sensing and the report of the local decision to the FC and opportunistic data transmission, respectively. The probability that the FC decides in favor of a free channel, $P_{\mathrm{free}}$, is given by:

(27) $$P_{\mathrm{free}}=P_{H_{1}}\left(1-P_{D,\mathrm{FC}}\right)+P_{H_{0}}\left(1-P_{\mathrm{FA},\mathrm{FC}}\right),$$

recalling that $P_{H_{1}}$ and $P_{H_{0}}=1-P_{H_{1}}$ are the probabilities that the PU transmitter is on and off, respectively, and $P_{D,\mathrm{FC}}$ and $P_{\mathrm{FA},\mathrm{FC}}$ are the global probabilities of detection and false alarm at the FC, respectively.

Notice from (26) that it is possible to reduce $E_{\mathrm{css}}$ by reducing the value of *M* in the first term, which means reducing the number of SUs that cooperate in the sensing process. However, this strategy may penalize the performance of the CSS. In [33,34], the authors propose ways of finding the optimal sensing interval and the optimal number of SUs in order to minimize energy consumption during the spectrum sensing phase, while satisfying the performance requirements for the CSS. The second term of (26) also increases with *M* and may represent a significant contribution to the total energy consumption. Finally, the energy consumption in the third term is dependent on the global performance of the CSS.

### 4.1. Energy Consumption of the Original Fusion Scheme

In the original fusion scheme of [26], the *M* secondary users perform local spectrum sensing in all frames, meaning that the first term of (26) is constant in all sensing rounds. Moreover, the energy consumption $ME_{R}$ in (26) is always equal to Mξ in this scheme, since the SUs transmit their local decisions in all frames via BPSK symbols having average energy *ξ*. Therefore, the average energy consumption per frame in the scheme of [26] becomes:

(28) $$E_{\mathrm{css}}=ME_{\mathrm{LS}}+M\xi +P_{\mathrm{free}}E_{T}.$$

### 4.2. Energy Consumption of the Pre-Compensated Fusion Scheme

In [28], the concept of the fusion of spectrum sensing information with pre-distorted transmissions is introduced, but no energy consumption analysis is made. This analysis is addressed in this subsection.

The energy consumption in the fusion scheme of [28] also follows (26). The consumption during local spectrum sensing, which is captured by the first term of (26), is the same as in [26], since in both schemes, all SUs perform local sensing in all frames. The energy consumption captured by the third term of (26), as already mentioned, depends on the global performance of the CSS. Thus, the main difference between these two schemes in terms of energy consumption resides in the second term of (26).

Recall from (4) that, to form the transmitted signal under the scheme of [28], each SU multiplies each BPSK symbol by a complex scalar that depends on the complex channel gain and on the clipping threshold. The average energy consumption of each SU during the report phase is then the value of ${\left|s_{k}\right|}^{2}$, averaged over all possible values of $\alpha_{k}$, for any *k*. Therefore, again assuming Rayleigh fading report channels, the average energy consumption of each SU during the report phase, $E_{R}$, becomes:

(29) $$\begin{array}{cc} E_{\mathrm{Rpc}} & =\int_{0}^{\infty }\min^{2}\left(\frac{1}{\alpha_{k}},C\right)\xi \frac{2\alpha_{k}}{\Omega }\exp\left(-\frac{\alpha_{k}^{2}}{\Omega }\right)d\alpha_{k}\\ & =E_{1}\left(\frac{1}{C^{2}}\right)\xi +C^{2}\left[1-\exp\left(-\frac{1}{\Omega C^{2}}\right)\right]\xi , \end{array}$$

where the additional subscript pc is a mnemonic for pre-compensated fusion, and $E_{1}\left(\cdot \right)$ denotes the generalized exponential integral function of order one, which is a built-in function in most mathematical software packages, such as MATLAB, Mathematica and Mathcad. It is worth mentioning that $E_{\mathrm{Rpc}}$ increases monotonically with the increase of *C*.

Then, the average energy consumption per frame in the scheme of [28] is:

(30) $$E_{\mathrm{css}}=ME_{\mathrm{LS}}+ME_{\mathrm{Rpc}}+P_{\mathrm{free}}E_{T}.$$

Equating $E_{\mathrm{Rpc}}$ to the average energy consumption for each SU as required by the original fusion scheme of [26], which is *ξ*, one can easily find that the clipping threshold is C≈1.12. Hence, for C<1.12, the energy consumption of the fusion scheme of [28] will be smaller than the one reached by the original fusion scheme of [26]. However, from [28], the values of *C* are often higher than 1.12. Therefore, the search for an strategy to reduce the energy consumption of the fusion scheme working under the idea of pre-compensation is completely justifiable. This is what is done by censoring the SUs’ pre-distorted transmissions, as demonstrated in the next subsection.

### 4.3. Energy Consumption of the Energy-Efficient Pre-Compensated Fusion Scheme

In the proposed fusion scheme, a given SU is allowed to transmit only if it declares the presence of the PU signal. Otherwise, it must remain silent. Therefore, (26) can be rewritten as:

(31) $$E_{\mathrm{css}}=ME_{\mathrm{LS}}+\bar{M}E_{\mathrm{Rpc}}+P_{\mathrm{free}}E_{T},$$

where $E_{\mathrm{Rpc}}$ is computed from (29), and $\bar{M}=Mp$ is the average number of SUs deciding in favor of the presence of the PU signal in each frame, with *p* being the probability $\mathrm{Pr}[m_{k}=1]$ given by (9). Therefore, while the average energy consumption during the report phase in the original fusion scheme of [26] and in the fusion scheme of [28] is fixed and given by Mξ and $ME_{\mathrm{Rpc}}$, respectively, it depends on $\bar{M}$ in the proposed scheme. The fist term in (31) has the same value for the schemes of [26,28] and for the new one.

### 4.4. Energy Efficiency

It is important to bear in mind that the strategy of censoring the SUs transmissions changes the decision rule with respect to those adopted in [26,28], which is an indication that the spectrum sensing performance would be affected. Moreover, one knows that the spectrum sensing performance affects the secondary network throughput due to its influence on $P_{\mathrm{free}}$, as can be concluded from (27). Therefore, the energy consumption of the three fusion schemes under analysis must be compared not only in terms of the energy expenditure during the report phase, but also during the opportunistic data transmission phase. The energy efficiency is the proper metric to address this comparison, as described in this subsection.

Recall that the secondary network can carry out an opportunistic transmission whenever it declares a vacant primary network band, an event that occurs with probability $P_{\mathrm{free}}=P_{H_{1}}\left(1-P_{D,\mathrm{FC}}\right)+P_{H_{0}}\left(1-P_{\mathrm{FA},\mathrm{FC}}\right)$. As a consequence, the secondary network can deliver its bits in a situation of low $P_{D,\mathrm{FC}}$, which is unfair from the primary network standpoint. Then, to address the energy efficiency in the secondary network, only those bits delivered when a free band is correctly identified must be considered. The term fair opportunistic transmission is used here to denote such a situation. Then, the probability of a fair opportunistic transmission is equal to the probability of correctly identifying an unused spectrum, which is given by $P_{H_{0}}\left(1-P_{\mathrm{FA},\mathrm{FC}}\right)$.

Assume that the opportunistic transmission interval is $T_{t}$, and that during this interval, the bit rate achieved by the SUs is $R_{b}$ bit/s. Then, the average number of bits fairly transmitted per frame is [18]:

(32) $$D=P_{H_{0}}\left(1-P_{\mathrm{FA},\mathrm{FC}}\right)R_{b}T_{t}.$$

The energy efficiency can be indirectly assessed by means of the amount of energy consumed by the whole secondary network per fair opportunistic transmitted bit [35] (p. 106). It is measured in joules per bit and computed as:

(33) $$Y=\frac{E_{\mathrm{css}}}{D},$$

where $E_{\mathrm{css}}$ is the total energy consumption of the secondary network, as given by (28), (30) and (31) for the fusion schemes proposed in [26,28] and for the new one, respectively. Thus, a more energy-efficient secondary network is the one that achieves lower values of Y.

## 5. Numerical Results and Discussions

The original fusion scheme proposed in [26] and the fusion scheme with pre-distorted transmissions suggested in [28] are identified by their reference numbers on the figures shown in this section. The proposed fusion scheme is denoted by the word new.

### 5.1. Spectrum Sensing Performance

Each value on the ROC curves presented hereafter was obtained from 500,000 Monte Carlo events. Each event corresponds to sending a zero-mean white Gaussian-distributed PU signal through *M* independent AWGN channels to the SUs. Without loss of generality, it is assumed that each SU makes its local decision on the PU signal activity by means of energy detection from N=100 received samples, for a given decision threshold *ζ*; see SubSection 3.1. The individual SUs’ decisions are sent to the FC through the report channels using a BPSK mapping or a censored transmission, depending on the fusion scheme under analysis. In the scheme of [26], BPSK symbols are transmitted to the FC in all report rounds. A similar procedure occurs when the scheme of [28] is applied, but the BPSK symbols are pre-distorted according to the channel gain, before transmission. In the new energy-efficient scheme, only the SUs that detect the presence of the PU signal send their pre-distorted symbols to the FC. The SUs’ decisions and the global decision at the FC are used separately for computing false alarm and detection rates, which are the estimates of the associated probabilities. This procedure is repeated by varying *ζ*, so that the ROC curves are traced-out.

**Figure 3:**
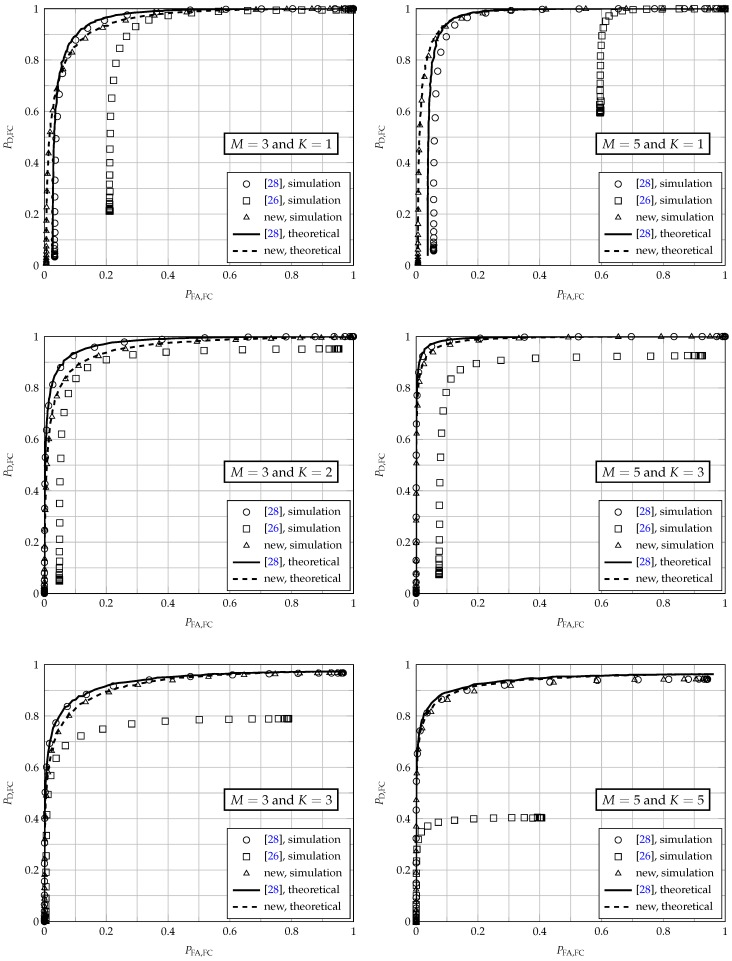
ROC curves at the fusion center for M=3 (left), M=5 (right), K=1 (top), K=⌈M/2⌉ (middle) and K=M (bottom).

Figure 3 shows the performances of the three fusion schemes under analysis, for M=3 and M=5 secondary users and for K=1, K=⌈M/2⌉ and K=M in the *K*-out-of-*M* rule. These values of *K* were chosen to configure the widely known decision fusion rules OR, majority-voting and AND, respectively. The received SNR at the SUs was arbitrarily set to $\gamma_{\mathrm{SU}}=-5$ dB, and the received SNR per bit at the FC was set to $\gamma_{\mathrm{FC}}=5$ dB. The report channel gains $h_{k}$ were drawn from a zero-mean complex Gaussian distribution with unitary second moment, thus representing flat and slow (constant during a report transmission) Rayleigh fading channels, with independent realizations from one report round to the next. The clipping threshold was arbitrarily chosen as C=3. The theoretical results regarding the fusion scheme of [28] are from [28] (Equations (23) and (24)). For the new scheme, they are from (23) and (24). The theoretical performances associated with the original fusion scheme are not shown, since the expressions reported in [26] only apply to AWGN report channels [27].

From the results shown in Figure 3, one can notice that the new scheme outperforms the original scheme of [26] in all cases under analysis. For M=5 and K=1, it also outperforms the scheme of [28]. In the remaining cases, the performance of the new scheme is close to the one of [28], which demonstrates that the combination of censoring and pre-distortion has not caused performance degradation with respect to the sole use of pre-distortion, yet bringing the advantage of less energy consumption. Moreover, it can be concluded that Expressions (23) and (24) are quite accurate for computing the probabilities of detection and false alarm achieved by the new scheme. The adherence between the theoretical results obtained from (23) and (24) and the empirical results is further explored in the Section 5.3.

### 5.2. Energy Consumption

The energy consumption analysis is firstly focused on the report phase; the other phases are considered later, by means of the energy efficiency parameter.

As previously mentioned, in the fusion schemes of [26,28], all SUs must transmit their local decisions to the FC in all report rounds. The energy consumption during the report phase is equal to Mξ for the scheme of [26], whereas it is equal to $ME_{\mathrm{Rpc}}$ for the scheme of [28]; see (28) and (30). From (29), it can be noticed that $E_{\mathrm{Rpc}}$ is dependent on the clipping threshold *C*. As an example, assume again E=ξ=1 and C=3. Then, the energy consumption during the report phase for the original scheme of [26] will be equal to *M*, whereas it will be equal to 2.6746M for the scheme in [28]. In this exemplifying situation, the energy consumption for the scheme proposed in [28] is almost three-times larger than the one attained with the original scheme.

**Figure 4:**
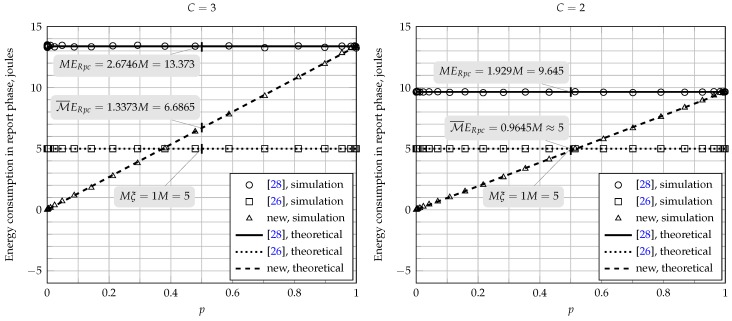
Energy consumption during the report phase for M=5, $\gamma_{\mathrm{SU}}=-5$ dB and E=ξ=1.

The proposed energy-efficient scheme introduces the concept of censoring in the report phase to reduce the problem of high energy consumption when the pre-compensation technique is applied. The energy consumption during the report phase now depends on the clipping threshold *C* and on the probability *p*, which in turn depends on the local sensing performance and PU signal activity, as can be retrieved from (9). For example, as can be observed from Figure 4 for E=ξ=1, C=3, $P_{H_{0}}=P_{H_{1}}=0.5$, $P_{D,\mathrm{SU}}=0.9$ and $P_{\mathrm{FA},\mathrm{SU}}=0.1$, the energy consumption of the CSS scheme during the phase report will be equal to 1.3373M, which is approximately 50% of the consumption produced by the fusion scheme of [28].

Figure 4 also shows other comparisons regarding the energy consumption during the report phase for the fusion schemes under analysis, assuming M=5, $\gamma_{\mathrm{SU}}=-5$ dB and E=ξ=1. Although the fusion scheme of [28] achieves substantially better performances when compared with those attained by the original scheme of [26] (see Figure 3), it can be seen from Figure 4 that the energy consumption of the former is considerably higher. On the other hand, the energy consumption of the new energy-efficient scheme depends on the local sensing performance. For instance, when C=3 and p=0.5 (from $P_{H_{0}}=P_{H_{1}}=0.5$, $P_{D,\mathrm{SU}}=0.9$ and $P_{\mathrm{FA},\mathrm{SU}}=0.1$), the energy consumptions are approximately 6.7 and five joules for the new scheme and for the original one, respectively. However, from Figure 3, it can be noticed that the new scheme always outperforms the original one. The energy consumption of the new scheme increases with *p*, but large values of *p* can be credited to high values of detection and false alarm rates in the local sensing processes, being the latter an undesired effect. The present behavior also happens when C=2, but the energy consumption of the new energy-efficient scheme and of the scheme proposed in [28] is lower, on average, when compared to the case of C=3. However, the performance is degraded as the clipping threshold decreases for both the new and the scheme of [28].

From above, one can conclude that a trade-off between performance and energy consumption must be established with respect to the clipping threshold, as demonstrated in the sequel.

### 5.3. Effect of the Clipping Threshold on Performance and Energy Consumption

**Figure 5:**
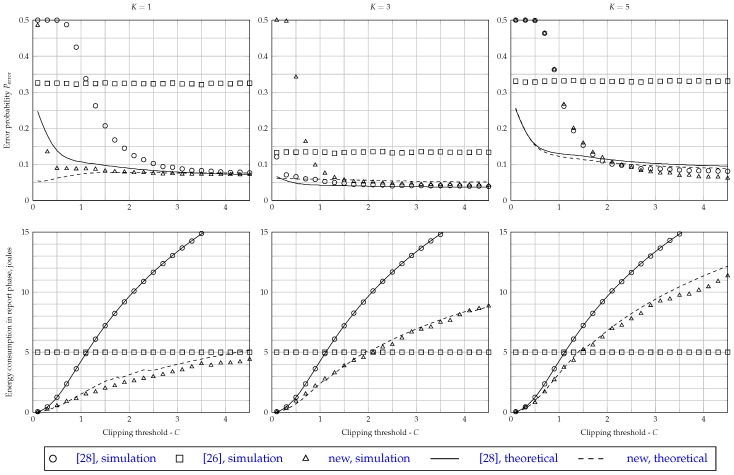
Error probability (top) and energy consumption (bottom) as a function of the clipping threshold *C*, for M=5, $\gamma_{\mathrm{SU}}=-5$ dB, $\gamma_{\mathrm{FC}}=5$ dB and E=ξ=1.

The clipping threshold *C* affects the energy consumption and the spectrum sensing performance of the proposed fusion scheme, as it does with respect to the one proposed in [28]. If *C* increases, the energy consumption increases, and the performance improves for both schemes. However, the performance improvement is not linearly proportional to *C*, as can be seen in the three top graphs of Figure 5, which consider M=5, $\gamma_{\mathrm{SU}}=-5$ dB, $\gamma_{\mathrm{FC}}=5$ dB and E=ξ=1. Notice that above a certain point, an increment in *C* produces a small reduction in the decision error probability at the fusion center. A decision error happens every time that the FC decides in favor of $H_{1}$ (or $H_{0}$), while it should have decided in favor of $H_{0}$ (or $H_{1}$). Therefore, the decision error probability can be expressed as:

(34) $$P_{\mathrm{error}}=P_{H_{0}}P_{\mathrm{FA},\mathrm{FC}}+P_{H_{1}}\left(1-P_{D,\mathrm{FC}}\right).$$

It is assumed that the best operating point on an ROC curve is the one corresponding to the pair ($P_{\mathrm{FA},\mathrm{FC}},P_{D,\mathrm{FC}}$), such that $P_{\mathrm{error}}$ is minimized for a given $P_{H_{0}}=1-P_{H_{1}}$.

It is worth emphasizing that the new parameter $P_{\mathrm{error}}$ was defined here to combine the performance metrics $P_{\mathrm{FA},\mathrm{FC}}$ and $P_{D,\mathrm{FC}}$ into a single one, thus facilitating the presentation of numerical results that consider the trade-off between the clipping threshold and the global reliability of the spectrum sensing.

Observe in Figure 5 that, for the considered decision combining rules OR (K=1), majority-voting (K=⌈M/2⌉=3) and AND (K=M=5), $P_{\mathrm{error}}$ is constant for any value of *C* in the case of the original fusion scheme of [26], since in this case, no clipping is applied.

For K=1 (upper-left graph of Figure 5), the error probability of the scheme of [26] is 0.33. It decreases as *C* increases for the scheme of [28]: for C<1.1, the performance of the scheme of [28] is worse than the one achieved by [26]; for C>1.1, the situation reverses. However, one can observe at the bottom-left graph of Figure 5 that the energy consumption of the scheme of [28] surpasses the one achieved with the original scheme of [26]. Since $P_{\mathrm{error}}$ remains practically constant for C≥3.1 in the case of the scheme of [28], C=3.1 can be considered a good clipping threshold. Therefore, in order to attain a good performance with the OR rule for the scheme of [28], the energy required is approximately 14 joules. On the other hand, the new energy-efficient scheme reaches the same performance with C≥0.5. According to Figure 5 (bottom-left graph), the energy consumption of the new fusion scheme operating under the OR rule and C=0.5 is equal to 0.493 joules, which is a value approximately 28-times smaller than what is consumed by the scheme of [28].

**Figure 6:**
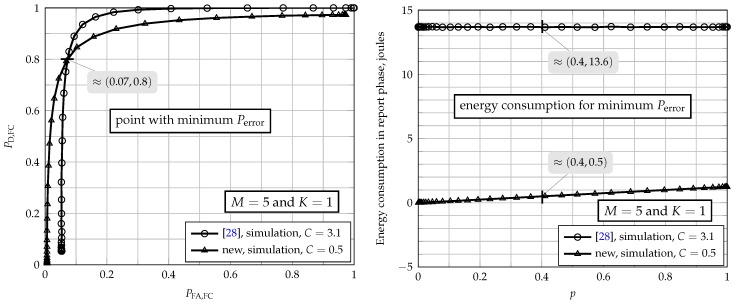
Performance (left) and energy consumption (right) for the new fusion scheme and for the one proposed in [28], for M=5, $\gamma_{\mathrm{SU}}=-5$ dB, $\gamma_{\mathrm{FC}}=5$ dB and E=ξ=1.

As an alternative view of a single result among those shown in the upper-left part of Figure 5, Figure 6 shows the performances of the fusion scheme of [28] and the new scheme, along with the corresponding energy consumptions during the report phase. Notice that C=3.1 was used in the former, while C=0.5 was used in the latter. There is a crossing point between the ROC curves of these two schemes, which corresponds to the same error probability for both. As can be noticed, at this crossing point, the energy consumption matches the one shown in Figure 5.

For the majority-voting rule, i.e., K=⌈M/2⌉=3, it can be observed from the upper-center graph of Figure 5 that the scheme of [28] outperforms the other two, since it achieves lower error probabilities. From the lower-center graph of Figure 5, it can be seen that, for C=0.5, the scheme of [28] yields low $P_{\mathrm{error}}$ with an energy consumption $ME_{R}\approx 5\times 0.25=1.25$ joules. The new scheme slightly outperforms the one of [26] for C>0.8. Moreover, the new scheme and the one of [28] achieves approximately the same performances for C>1.5, although the new one spends less energy.

When the AND decision fusion rule (K=M=5) is considered, it can be noticed from the upper-right graph of Figure 5 that both the new scheme and the one of [28] achieve practically the same $P_{\mathrm{error}}$ for any value of *C*, outperforming the original scheme for C>1, approximately. In this case, from the and bottom-right graph of Figure 5, one can observe that the new scheme is more efficient than the one of [28], since it attains lower energy consumption.

Still referring to Figure 5, it can be concluded that the expressions (23) and (24) for computing of the global spectrum sensing performance are capable of producing good adherence with the simulation results for the values of the clipping threshold *C* that produce accurate approximations of the probability density functions used to derive these expressions. Notice that the performances obtained from simulations considering the OR and the majority-voting rules are quite close to the theoretical results for C>1.5, while for the AND rule, the theoretical results become close to the simulation results for C>1.9. For lower values of the clipping threshold, the theoretical expressions diverge from the simulation results, which is an expected effect. The expressions derived in this paper regarding energy consumption, (30) and (31), were also found to achieve good agreement with the simulation results for all values of *C* for both schemes that are influenced by *C*, which are the new scheme and the one proposed in [28]. Quite similar behaviors were observed for other values of *M*, but with different numerical results. These results were omitted for conciseness.

It is important to mention that the results presented in Figure 5 are in agreement with the observations made in [28] (p. 10903), where it is shown that, for M=5 and K=1, the adherence between theoretical and simulated spectrum sensing performance results occurs for C>3.7.

### 5.4. Energy Efficiency

**Figure 7:**
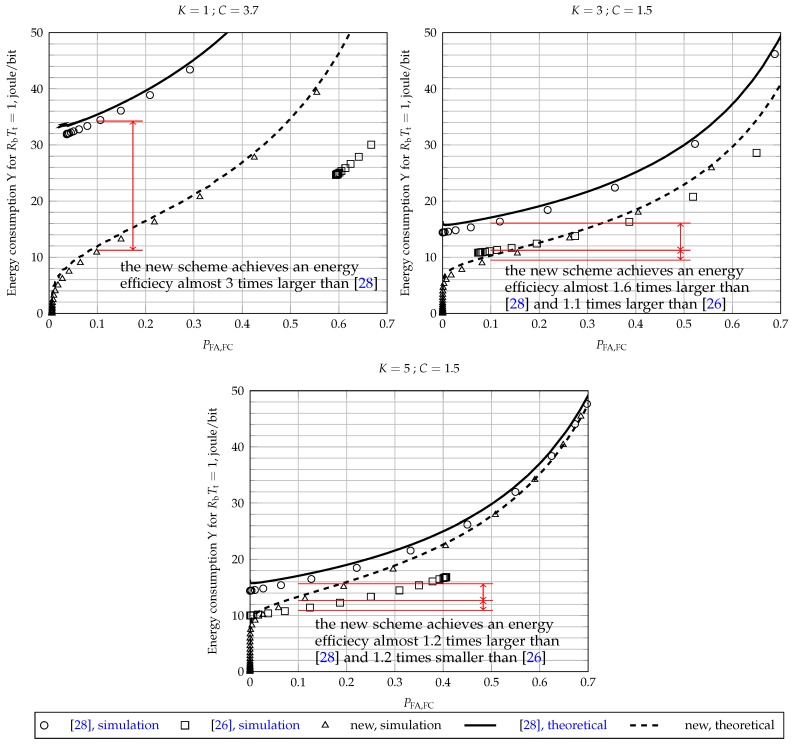
Energy consumption per $R_{b}T_{t}$ fair opportunistic transmitted bits, for M=5, K=1 (top-left), K=3 (top-right) and K=5 (bottom), $\gamma_{\mathrm{SU}}=-5$ dB, $\gamma_{\mathrm{FC}}=5$ dB and E=ξ=1.

The graphs in Figure 7 allow for the assessment of the energy efficiencies achieved by the three fusion schemes under analysis. They show the amount of energy consumed by the whole secondary network per fair opportunistic transmitted bit, assuming without loss of generality that $R_{b}T_{t}=1$; see (32) and (33). Thus, the consumption per fair transmitted bit of a real network is obtained by simply dividing the consumptions shown in these graphs by the actual value of $R_{b}T_{t}$. The system parameters considered to draw Figure 7 are M=5, K=1 (OR rule), K=3 (majority-voting rule) and K=5 (AND rule), $\gamma_{\mathrm{SU}}=-5$ dB, $\gamma_{\mathrm{FC}}=5$ dB and E=ξ=1. The clipping thresholds were chosen in order to approximate the decision error probabilities of new scheme and the scheme of [28] and were obtained from the three upper graphs of Figure 5: C=3.7 for K=1, C=1.5 for K=3 and C=1.5 for K=5.

It can be verified from the top-left graph of Figure 7 that the new fusion scheme achieves an energy efficiency considerably higher (considerably lower consumption per fair opportunistic transmitted bit) than the other two schemes when K=1, for values of $P_{\mathrm{FA},\mathrm{FC}}$ of practical importance. For instance, at $P_{\mathrm{FA},\mathrm{FC}}=0.1$, the secondary network consumes Y≈12 joules per $R_{b}T_{t}$ fair opportunistic transmitted bits. For the same false alarm probability, the scheme of [28] consumes Y≈34 joules per $R_{b}T_{t}$ bits, that is an energy efficiency three-times smaller than the new scheme. The original scheme of [26] it is not capable of reaching $P_{\mathrm{FA},\mathrm{FC}}=0.1$ for the system parameters under consideration; the lowest value of $P_{\mathrm{FA},\mathrm{FC}}$ is around 0.57, which is useless in practice. In this situation, the energy cost to transmit $R_{b}T_{t}$ bits opportunistically is Y≈25 joules.

In the case of K=3, one can observe in the top-right graph of Figure 7 that the energy efficiency of all schemes improved in comparison to the case of K=1; the most expressive improvement was achieved by the scheme of [28]. Moreover, it can be noticed that the energy efficiency of the new scheme is now comparable to the one attained by the original fusion scheme of [26], remaining better than the one achieve by the scheme of [28]. The energy consumption of the proposed scheme is Y≈10 joules per $R_{b}T_{t}$ transmitted bits for $P_{\mathrm{FA},\mathrm{FC}}=0.1$, whereas Y≈11 and Y≈16 joules per $R_{b}T_{t}$ bits for the schemes of [26,28], respectively. Therefore, the new scheme achieves an energy efficiency almost 1.6-times larger than [28] and 1.1-times larger than [26].

For K=M, one can notice from the graph on the bottom of Figure 7 that the new scheme achieves a slightly better energy efficiency than the scheme of [28] for any value of $P_{\mathrm{FA},\mathrm{FC}}$ and a slightly worse energy efficiency than the scheme of [26] for $P_{\mathrm{FA},\mathrm{FC}}>0.02$, approximately. The new scheme wins over the other two for $P_{\mathrm{FA},\mathrm{FC}}$ below 0.02, approximately. For $P_{\mathrm{FA},\mathrm{FC}}=0.1$, the new scheme achieves an energy efficiency almost 1.2-times larger than [28] and 1.2-times smaller than [26].

**Figure 8:**
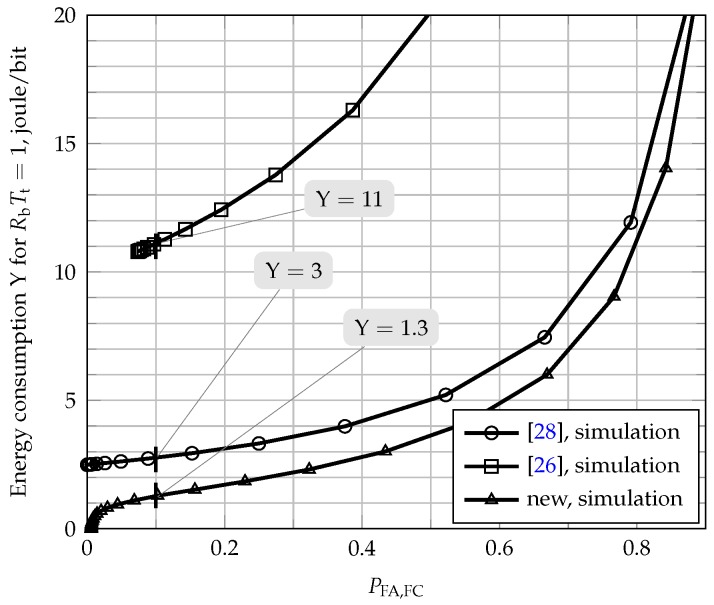
Lowest energy consumption per $R_{b}T_{t}$ fair opportunistic transmitted bits (highest energy efficiency), for M=5, K=1 and C=0.5 for the new fusion scheme, K=3 and C=0.5 for the scheme of [28] and K=3 for the scheme of [26].

Finally, the higher energy efficiencies that can be reached by the three analyzed schemes are compared by means of Figure 8. Based on the simulation results shown in the upper graphs of Figure 5, for each scheme, it is considered the smallest value of the clipping threshold *C* beyond which no expressive variation in the smallest $P_{\mathrm{error}}$ is perceived. Therefore, the better energy efficiency of the proposed scheme is for K=1 and C=0.5, while it happens for K=3 and C=0.5 in the scheme of [28] and for K=3 in the original scheme of [26]; recall that [26] does not apply clipping to the transmitted report signals. It can be concluded from Figure 8 that the energy efficiency of the new scheme outperforms the other two. For instance, again assuming $P_{\mathrm{FA},\mathrm{FC}}=0.1$, the energy consumed per $R_{b}T_{t}$ transmitted bits is Y≈1.3 joules for the new scheme, being Y≈3 joules for the scheme of [28]. The original scheme of [26] has a quite lower energy efficiency, reaching to Y≈11 joules per $R_{b}T_{t}$ fair opportunistic transmitted bits in its best configuration. Therefore, for the best energy efficiency configurations, the new scheme achieves an energy efficiency almost 2.3-times larger than [28] and 8.5-times larger than [26].

In order to illustrate the practical significance of the results presented in Figure 8, consider that the opportunistic transmission interval is $T_{t}=40$ ms and that the bit rate achieved by each SU is $R_{b}=10$ kbit/s [35] (p. 111). In this scenario, the network energy efficiency is $Y=1.3/R_{b}T_{t}=1.3/400=3.25\times 10^{-3}$ joules per bit opportunistically transmitted. Following the same reasoning, the energy efficiency of the schemes of [26,28] is respectively $Y=11/400=27.5\times 10^{-3}$ and $Y=3/400=7.5\times 10^{-3}$ joules per bit opportunistically transmitted. Therefore, in this scenario of highest energy efficiency, the new scheme has an energy consumption per transmitted bit 57% smaller than the scheme of [28] and 88% smaller than the original scheme of [26]. This example shows that the proposed fusion scheme allows the establishment of a good trade-off solution between bandwidth efficiency and energy consumption for cooperative spectrum sensing networks.

## 6. Conclusions and Final Remarks

A novel scheme for the fusion of spectrum sensing information in cooperative spectrum sensing for cognitive radio applications was proposed in this paper. The scheme combines a spectrum-efficient, pre-distortion-based fusion strategy with an energy-efficient censoring-based fusion strategy to achieve the combined effect of reduction in bandwidth and power consumption during the phase of reporting the spectrum sensing decisions to the fusion center.

Expressions for computing the key performance metrics of the spectrum sensing of the new fusion scheme were derived and validated by means of computer simulations. It has been shown that the theoretical results are quite accurate for values of the number of cooperating secondary users and clipping thresholds of practical significance.

An extensive analysis of the overall energy efficiency was also made for the proposed scheme, along with comparisons with related references proposed in the literature. It has been demonstrated that the proposed fusion scheme can outperform the energy efficiency attained by those reference strategies, while it attains the same spectrum efficiency and approximately the same global decision performance of the best among those strategies.

The scheme proposed in this paper plays a special role in spectrum sensing techniques for cognitive radio applications, which is the energy and bandwidth saving during the transmission of the spectrum sensing decisions through the resource-limited control channel, without significant performance loss. Moreover, since the knowledge of the channel state information can be obtained by the fusion center only once within the channel coherence time and because this scheme provides higher spectrum efficiency, it can be used in mobile nodes with reduced impact on the secondary network performance.

The performance of the proposed fusion methodology was accessed assuming flat and slow fading channels between the secondary users and the fusion center, corrupted with additive Gaussian noise, which represents a general enough scenario to demonstrate the feasibility of the methodology. Nevertheless, more elaborated models could be adopted, for instance considering generalized fading, frequency and time selectivity, as well as other sources of impairments, such as impulsive noise and intentional and non-intentional interference. The proper operation of the proposed methodology in these new scenarios is unknown and represents a formidable research opportunity. Moreover, one must recall that the actual performance of the secondary network in terms of throughput and controlled interference to the primary network is also influenced by the reliability of the fusion center transmissions that will eventually control the opportunistic transmissions of the secondary users. Packet losses due to collisions or channel impairments and timing coordination imperfections are examples of problems that may occur in real networks, but affect the performance of any spectrum sensing and dynamic access.

## Abbreviations

The following abbreviations are used in this manuscript:

AWGN	Additive white Gaussian noise
BPSK	Binary phase-shift keying
CR	Cognitive radio
CSS	Cooperative spectrum sensing
ED	Energy detection
FC	Fusion center
ML	Maximum likelihood
PAPR	Peak-to-average power ratio
PDF	Probability density function
PU	Primary user
ROC	Receiver operating characteristic
SU	Secondary user
